# Supplementary material for: Association between oral microbiome and seven types of cancers in East Asian population: a two-sample Mendelian randomization analysis
Source: Front Mol Biosci. 2023 Nov 21;10:1327893. doi: 10.3389/fmolb.2023.1327893 (PMC10702768; doi:10.3389/fmolb.2023.1327893)
Supplement: Supplementary file 1 [file DataSheet1.ZIP › Supplementary Materials/MR plots of saliva/saliva═╝/Lung Cancer/pheno.858_to_lung cancer_forest.pdf]

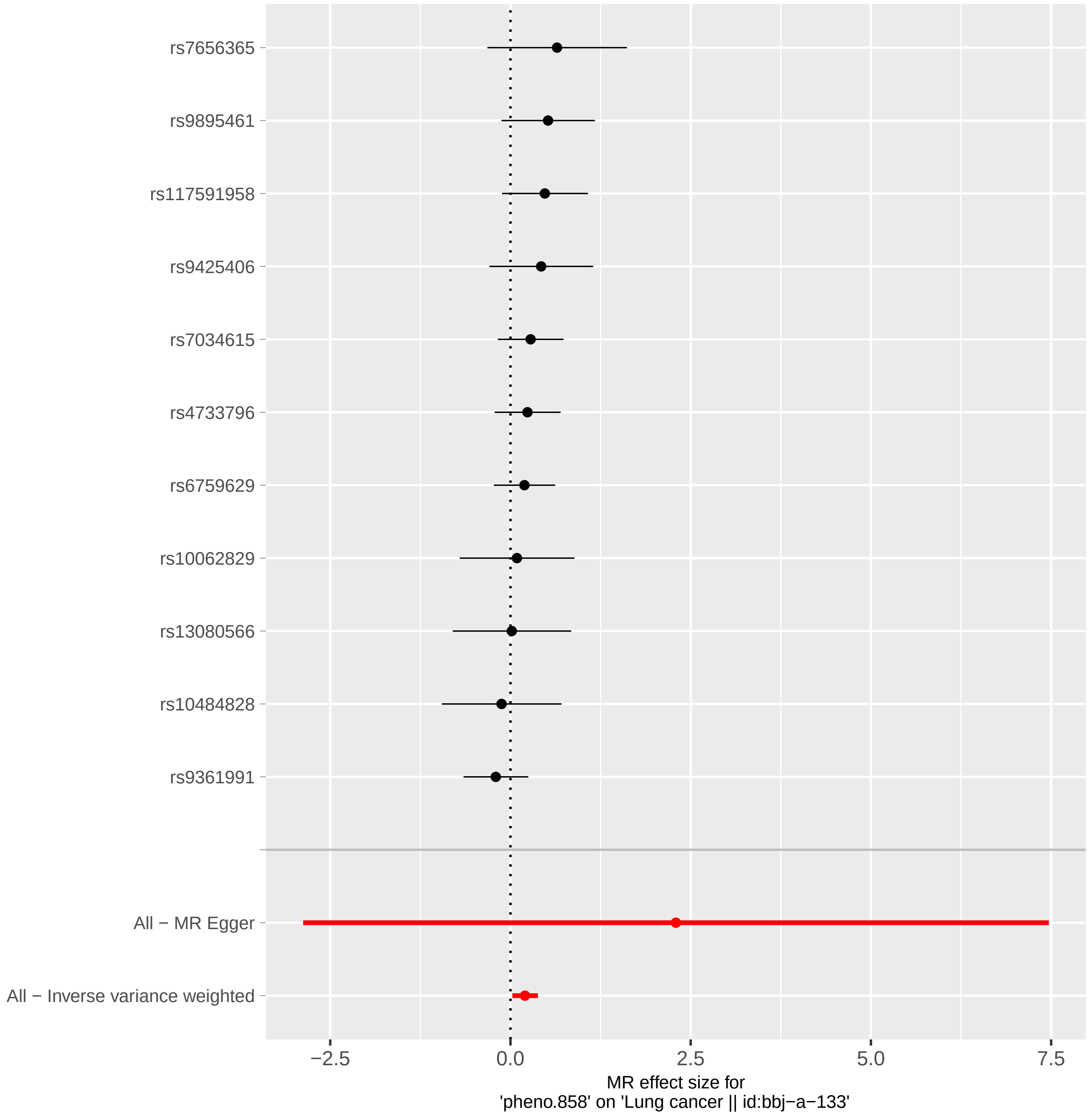
rs7656365
rs9895461
rs117591958
rs9425406
rs7034615
rs4733796
rs6759629
rs10062829
rs13080566
rs10484828
rs9361991
All − MR Egger
All − Inverse variance weighted
−2.5
0.0
2.5
5.0
7.5
MR effect size for
'pheno.858' on 'Lung cancer || id:bbj−a−133'
